# In-vitro and in-vivo respiratory deposition of a developed metered dose inhaler formulation of an anti-migraine drug

**DOI:** 10.1080/10717544.2019.1618419

**Published:** 2019-07-05

**Authors:** Ebtsam M. Abdou, Soha M. Kandil, Amany Morsi, Maysa W. Sleem

**Affiliations:** a Department of Pharmaceutics, National Organization of Drug Control and Research (NODCAR), Giza, Egypt;; b Department of Pharmaceutics and Industrial Pharmacy, Faculty of Pharmacy, MTI University, Cairo, Egypt;; c Department of Analytical Chemistry, National Organization of Drug Control and Research (NODCAR), Giza, Egypt;; d Research and Development, ADCO Pharmaceutics Co, Cairo, Egypt

**Keywords:** Anti-migraine, HFA propellant, metered dose inhaler, next generation impactor, zolmitriptan

## Abstract

Enhancement of zolmitriptan bioavailability through development of micronized zolmitriptan pressurized metered dose inhaler (MDI) as an alternative to its traditional drug delivery systems. A reversed phase HPLC method for zolmitriptan determination was developed and evaluated. Micronized zolmitriptan MDI formulations were prepared using two different propellants. The prepared formulations were evaluated for mean shot weight, drug content, and leakage rate in addition to in-vitro deposition using next generation impactor where mass median aerodynamic diameter (MMAD), geometric standard deviation (GSD), fine particle dose, fine particle fraction (FPF), emitted dose (ED), and dispersibility were determined. The selected formulation was evaluated for in-vivo bronchial absorption in rats. The physicochemical characters of the prepared formulations were found to be dependent mainly on the vapor pressure of the used propellant. MDI formulation prepared with HFA 134a propellant was found to have the lowest MMAD (3.47 ± 0.65) with GSD of 2.3 ± 0.4. It also had the highest FPF (41.9), ED (89.26 ± 2.35) with dispersibility of 46.9%. This formulation, when applied to rats, resulted in faster *T*_max_ (27 ± 5 min) with higher *C*_max_ (1236 ± 116 ng/mL) and AUC_(0-12)_ (3375 ± 482 ng/mL·h) over the oral tablet. Its relative bioavailability was 72.7% which was 1.25 times higher than the oral tablet relative bioavailability. Zolmitriptan MDI formulation was developed using micronized zolmitriptan powder without further modification or particle engineering. The developed formulation using HFA 134a propellant could be favorable alternative, with enhanced bioavailability, to zolmitriptan oral tablet for acute migraine treatment.

## 1. Introduction

Pharmaceutical development of new drug formulations and delivering them via different portals into the body to enhance drug bioavailability is the main concept of recent pharmaceutical research. Among these developments, systemic drug delivery via the pulmonary route has recently gained great interest. The pulmonary rout is characterized by being an attractive noninvasive administration route with lower dose of drug required to achieve a therapeutic effect in comparison to oral administration due to its high vascular nature, large surface area, and avoidance of the first pass effect (Pilcer & Amighi, 2010; Patil & Sarasija, 2012; Stank & Steckel, 2013). Traditionally, the pulmonary drug delivery was restricted to localized pulmonary diseases treatment such as asthma and other airways diseases. But, nowadays, there is a growing interest to use the pulmonary drug absorption for systemic drug delivery of different drugs such as insulin (Hamishehkar et al., 2010; Al-Qadi et al., 2012; Zhao et al., 2012) with a nowadays marketed product; Exubera^®^ marketed at Pfizer, low molecular weight heparin (Yang et al., 2006; Bai & Ahsan, 2010; Patel et al., 2012), hepatitis B vaccine (Lombry et al., 2004; Thomas et al., 2008), and some antibiotics such as tobramycin, aztreonam, and ciprofloxacin (Hickey, 2013). For treatment of acute migraine, inhaled formulations of dihydroergotamine, prochlorperazine (PCZ), and loxapine were developed (Chua & Silberstein, 2016).

The pressurized metered dose inhaler (MDI) is one of the oldest and most commonly prescribed therapeutic systems for drug delivery to the lungs (Stein et al., 2014). The key components of the MDI are the container, propellant, concentrated drug formulation, metering valve, and actuator. All play roles in the formation of the aerosol cloud and delivery efficiency (Smyth, 2003; Newman, 2005). They mainly depend on using different types of propellants but recently, hydrofluroalkane (HFA) propellant-based MDI formulations were developed to be used instead of chlorofluorocarbons (CFC) propellants-based formulations which were banned since 1996 as they were reported to destroy the ozone layer (Talasila et al., 2013). MDI formulations are prepared either by dissolving the drug into the HFA (such as HFA 134a and HFA 227) if it has sufficient solubility in the HFA, or using a co-solvent, usually ethanol, to increase the solubility of the drug in the HFA. In case that the drug has no solubility in the HFA, it is prepared as a micron-sized suspension in HFA (Pritchard, 2001; Ooi et al., 2015). Solution MDI formulations are relatively easy to be formulated in comparison to suspension formulations which suffers from tendency to flocculate and have a potential for physical instability (Khale & Bajaj, 2011) although they were reported to reduce bad tastes of the drug (Hickey, 2002). But due to insolubility of many drugs in HFA propellant, suspension formulations development is worthy explored.

Migraine headache is the most common neurological vascular headache disease which causes a throbbing and pulsating pain around the head due to brain and scalp arteries dilating resulting in terrible pain in the head (Green et al., 2005). Migraine treatment usually requires the drug to be accessed through the systemic circulation as the target tissue here, which is the brain, cannot be accessed through local therapy. So, rapid drug delivery to the circulation is required (Misra et al., 2003). Studies have shown that migraine patients consider rapid onset, complete and lasting pain relief, and medication side effects as the most important factors when choosing a migraine treatment (Gallagher, 2004).

Zolmitriptan (4S-4-({3-[2-(dimethylammino)ethyl]-1H-indol- 5-yl}methyl-1,3-oxazolidin2-one) is second generation triptan which is an effective, well-tolerated treatment of acute migraine associated with menses, migraine with aura. It has a selective action on serotonin (5HT1B/1D) receptors and is very effective in reducing migraine symptoms. It is also effective in treatment of acute cluster headache (Cittadini et al., 2006; Cortelli et al., 2017). Oral administration zolmitriptan has been reported to show slow onset of action, low bioavailability (40%), nausea, and incomplete pain relief with recurrence of headaches (Ahonen et al., 2004; Goadsby & Yates, 2006; Mittal et al., 2014) with considerable ratio of migraine patients suffering from gastric stasis, severe nausea, and vomiting during the migraine attack. The matters which causes erratic absorption of the drug from the gastrointestinal tract with delayed gastric emitting which makes the oral treatment is ineffective (Vyas et al., 2005; Aurora et al., 2007; Egla & Abd Al hammid 2016).

The aim of this study was to develop micronized zolmitriptan MDI formulations using different propellants. The formulations were evaluated in-vitro for their airway deposition. The best formulation was evaluated in-vivo in rats for its bronchial absorption.

## 2. Methodology

### 2.1. Materials

Micronized zolmitriptan and oleic acid were purchased from Sigma Aldrich (Merch), Germany. HFA 134a and HFA 227 were purchased from Mexichem Co., United Kingdom. HPLC grade acetonitril and other analytical reagents were purchased from El-Gomheria Co., Cairo, Egypt.

### 2.2. Particle size distribution of micronized zolmitriptan dry powder

The particle size of micronized zolmitriptan was measured using a laser-diffraction particle size distribution analyzer (LA-950, Horiba Ltd., Kyoto, Japan) using n-hexane as a suspending medium at circulation speed (5) and agitation speed (2) continuous with no sonication. The experiment was done three times and the mean diameter (*D*), median, *D* (10%), (50%), and (90%) were determined. The span was calculated from the following equation:

$$\mathrm{Span}=\frac{D\;\left(90\%\right)-D\;(10\%)}{D\;(50\%)}$$

### 2.3. Development of HPLC method for zolmitriptan determination

For zolmitriptan determination method, the following HPLC system was used: HPLC system, Dinox UltiMate3000 equipped with a variable wavelength diode array detector, a quaternary pump and a manual injector 20 µl loop (USA). Hamilton syringe 10 µl capacity. Chromatographic separation was achieved on Kromacil C-18,150 × 4.6 mm, 5 µ column and isocratic elution with sodium dihydrogen orthophosphate buffer (pH 6.8): acetonitrile (60:40). The detection wavelength was 225 nm, flow rate of 1 ml/min, injection volume 20 µl, column temperature 30 °C. To prepare the phosphate buffer, 6.8 g of potassium dihydrogen orthophosphate were accurately weighed using electric balance (Shimadzu, Japan), transferred into 1000 ml volumetric flask, dissolved in and the volume was completed with water. pH of the buffer was adjusted using 0.0 M sodium hydroxide solution (pH meter, HANA9321 Microprocessor, Portugal).

#### 2.3.1. Preparation of standard solution

Zolmitriptan stock solution was prepared by dissolving 25 mg zolmitriptan in 10 ml mobile phase (buffer: acetonitrile (60:40)) into 25 ml volumetric flask and then the volume was completed with the same solution. Serial dilutions of zolmitriptan were prepared from the stock solution to obtain required working standard concentrations.

#### 2.3.2. Preparation of sample solutions

*Procedure for metered dose assay sample:* Remove all labels and markings present on the container with acetonitrile, prime it with three actuations. Wash the valve with methanol and wipe with tissue paper. Shake the pressurized container for 20 s and place it inverted in 50 ml beaker containing 30 ml of mobile phase (buffer:acetonitrile (60:40)) and release one actuation. Repeat the sequence for 10 times, transfer into 250 ml volumetric flask, rinse the beaker with mobile phase, add rinsate to the 250 ml volumetric flask and make up to mark with mobile phase.

#### 2.3.3. Method validation

The developed method was validation as per ICH guideline (ICH-Guidelines). The validation parameters are linearity, accuracy, precision, and robustness.

##### 2.3.3.1. System suitability

The HPLC system was stabilized for 30 min by passing the mobile phase at the determined wave length and flow rate to get a stable base line. The six replicated injections were made, after one blank injection, in the standard solutions of zolmitriptan. System suitability parameter such as theoretical plates (USP), retention time, relative standard deviation, and tailing factor were evaluated.

##### 2.3.3.2. Linearity

Linearity was demonstrated from seven different concentration levels for zolmitriptan which were found to be linear in the range of 4–60 µg/ml (4, 10, 20, 30, 40, 50, and 60 µg/ml).

##### 2.3.3.3. Accuracy (recovery studies)

To check the degree of accuracy of the method, the recovery studies were performed by the developed method at different concentrations within linearity range, 15, 25, and 35 µg/ml, were analyzed in triplicates and the mean recovery % was calculated.

##### 2.3.3.4. Precision

Intraday precision (repeatability) and interday precision (reproducibility) were evaluated by carrying out four independent sample preparations and the percentage relative standard deviation (%RSD) was calculated.

##### 2.3.3.5. Robustness

To evaluate the robustness of the developed HPLC method, small deliberate variations in the parameters of optimized method were done, ±5 °C change in temperature, ±0.15 ml change in flow rate, the retention time, and area were studied.

##### 2.3.3.6. LOD and LOQ

The limit of detection (LOD) and limit of quantification (LOQ) were calculated according to the following equations: LOD = 3.3σ/*S*; LOQ = 10σ/*S*, where σ = the standard deviation of the response; and *S* = the slope of the calibration curve.

### 2.4. Formulation of HFA-based zolmitriptan MDI formulations

Zolmitriptan MDI formulations were prepared using two propellants: HFA 134a (F1), HFA 227 (F2) and 1:1 mixture of them (F3). 1% w/w oleic acid was used as-stabilizer. In addition, 1% w/w of dried ethanol was used to improve the solubility of the stabilizing agent and to enhance valve function. Zolmitriptan MDI formulations were prepared using pressure filling technique. Micronized zolmitriptan (0.6 g) and oleic acid were weighed into clean 15-ml canisters and dispersed into the dried ethanol. The canisters were immediately crimped with metering valve 50 µl (V.A.R.I., Italy) using Pamasol aerosol crimping machine with 20 mm neck diameter crimping collet. The propellant was then added from a pressure buret attached to the filling machine to bring the final weight in each vial to 12 g. The complete formulation is then fired under pressure into the canister. To ensure complete dispersion of the drug powder in the propellant, the canisters were sonicated for 60 s in an ultrasonic bath (Sonifier 250 Bransan, Germany) and then they were placed onto platform shaker (GYROMAX 800-Series Shaker, Amerex Instruments, USA) at 150 rpm and allowed to shake for 24 h.

### 2.5. Evaluation of the prepared zolmitriptan MDI formulations

#### 2.5.1. Determination of mean emitted shot weight (delivered amount)

To determine the shot weight, the first five actuations were fired in air and then the canister was first separated from the adaptor, weighed and the weight was recorded (*W*_1_). The canister was placed back into its actuator. Ten successive actuations were sprayed from the inhaler with 5 s difference between each actuation and the other one. The canister was subsequently removed from the adaptor, the valve stem and the orifice were wiped clean. The canister was weighed again and the weight was recorded (*W*_2_) (Khale & Bajaj, 2011).

Average weight per metered dose = *W*_1_–*W*_2_/10.

The test was performed in triplicates for each formulation from three positions (first, middle, and last actuations) and the average weight was reported.

#### 2.5.2. Uniformity of the delivered and emitted dose drug content

The zolmitriptan content of the delivered actuation was determined by agitating the canister for 30 s and keeping it in inverted position into a beaker containing diluent (phosphate buffer:acetonitrile (60:40)). The inhaler was discharged in the inverted position under the surface of the solvent. The first actuations were fired out, then the first 10, middle 10, and on the last 10 deliveries were collected from the container (Sukasamea et al., 2011). The drug content of these deliveries was estimated using the above developed HPLC method.

#### 2.5.3. Total number of discharges per canister

It was done by counting the number of priming discharges at intervals of not less than 5 s until the canister was empty. The experiment was done three times for every formulation and the average number was recorded (Uddin et al., 2016).

#### 2.5.4. Propellant leakage rate determination

It was determined by selecting 12 canisters. To check any leak from the containers, they were kept in a water bath maintained at 50 °C. After equilibration, canisters are checked for the presence of any leaks in the form of air bubbles arising from the orifice or the valve crimp. Each canister was weighed (*W*_1_) in mg with reporting the date and the time to the nearest half hour. The canisters were allowed to stand in an upright position at temperature of 25 ± 2° for not less than 3 days (Shrikhandea et al., 2011; USP 29a); in our experiment canisters were left for 14 days. After a period of 14 days, the canisters were reweighed separately and the weight was recorded (*W*_2_) in mg with reporting the date and time to the nearest half hour. The leakage rate in mg/year was calculated from the following formula:

$$\text{Mg loss per year}=\frac{365\times 24\times \left(W_{1}-W_{2}\right)}{T}$$

$$\%\;\mathrm{Leakage}=\frac{\left(\frac{\mathrm{Mg}}{\mathrm{year}}\right)\times \;100}{\text{Net fill weight}}$$

#### 2.5.5. Moisture content determination

Moisture content of the prepared MDI formulations was determined using Karl Fisher Coulometer (Metrohm Ltd, Switzerland). The canister was weighed, shaken and then attached to the adaptor in the titration cell of the Coulometer. The canister was actuated through the induction port five times and on the fifth actuation the canister was held down for 10 s before release. The canister was weighed and the weight of the five actuations was calculated and transferred directly into the Coulometer software by RS232 connection from the sample size. The water content was automatically quantified (Abdul Motalib et al., 2012).

#### 2.5.6. Microbial contamination determination

It was determined by suspending 1 g of the canister contents to be examined in tryptone soya broth not to have antimicrobial activity then diluting the volume to 10 ml then homogenizing the suspension mechanically (USP 29b).

##### 2.5.6.1. Plate count method for bacteria

It was determined by using sterile petri dishes 9–10 cm diameter, then spreading 0.1 ml of prepared suspension on the surface of solidified medium in petri dishes (15 ml liquefied casein soyabean digest agar) at not more than 45 °C, then the petri dishes were incubated at 30 °C–35 °C for 5 days.

##### 2.5.6.2. Plate count method for fungi

It was determined by using sterile petri dishes 9–10 cm diameter, then spreading 0.1 ml of prepared suspension on the surface of solidified medium in petri dishes (15 ml liquefied sabouraud dextrose agar) at not more than 45 °C, then the petri dishes were incubated at 20 °C–25 °C for 5–7 days.

##### 2.5.6.3. Pathogenic organism (*Pseudomonas aeruginosa*) determination

It was determined by inoculating 100 ml of casein soya bean digest broth with 10 ml of prepared suspension to be examined then, the mixture was incubated at 35 °C–37 °C for 24–48 h then on plate of citrimide agar subculturing and incubating at 35 °C–37 °C for 24–48 h.

##### 2.5.6.4. Pathogenic organism (*Staphylococcus aureus*) determination

It was determined by inoculating 100 ml of casein soya bean digest broth with 10 ml of prepared suspension to be examined then, the mixture was incubated at 35 °C–37 °C for 24–48 h then on plate of Baird parker agar subculturing and incubating at 35 °C–37 °C for 24–48 h.

### 2.6. In-vitro MDI deposition studies using the next generation impactor (NGI)

The in-vitro aerodynamic parameters of the prepared zolmitriptan MDI formulations were assessed using the NGI with pre-separator and USP induction port (Copley Scientific, Nottingham, UK). The NGI was assembled and operated in accordance with USP General Chapter 601 to assess the drug delivered.

#### 2.6.1. NGI setup

To ensure efficient particle capture and prevent inter-stage losses due to particle bounce, the cups were coated with 2% poly ethylene glycol 400 in acetone using spray gun and placed under the fume hood until the complete evaporation of acetone. The mouth piece adaptor was attached to NGI induction port and the vacuum source was attached to the exhaust port of the impactor. The flow rate was calibrated using a flow meter (DFM 2000, Copley Scientific, Nottingham, UK) and the airflow was adjusted to 30 l/min (±5%) in order to model the flow rate in healthy adult lung (Abdelrahim & Chrystyn, 2009).

#### 2.6.2. Releasing dose for particle size analysis

The vacuum pump was turned on and was allowed for 1 min to stabilize the airflow before inserting actuator into the mouthpiece adaptor on the induction port of NGI. The device was actuated for once keeping the valve depressed for 5 s. The vacuum pump was turned off between actuations allowing the airflow to reach zero before restarting again. The airflow was stabilized before re-inserting the actuator and the canister was shaken for 10 s before the next actuation. The process was repeated 9 times so that the totals of 10 actuations were collected within NGI. Mass median aerodynamic diameter (MMAD) and geometric standard deviation (GSD) indexes were calculated. The MMAD is defined as the diameter at which 50% of the particles by mass are larger and 50% are smaller (Aghdam et al., 2016).

#### 2.6.3. Sample collection from actuator

The actuator was removed after the last actuation from mouthpiece adaptor. The actuator was rinsed by 20 ml zolmitriptan mobile phase (phosphate buffer: acetonitrile (60:40)) in 25 ml volumetric flask then completing volume to 25 ml using the same diluent. Zolmitriptan content was determined through injection into the HPLC system.

#### 2.6.4. Sample collection from the induction port

The induction port was removed after the last actuation. The induction port was rinsed using 10 ml zolmitriptan mobile phase. The washing was repeated four times then completing volume to 50 ml in volumetric flask using zolmitriptan mobile phase. Zolmitriptan content was determined through injection into the HPLC system.

#### 2.6.5. Sample collection from the collection cups

After removing the inhaler from the mouthpiece adaptor and the induction port after the last actuation, the collection cups were removed and rinsed by adding 10 ml zolmitriptan mobile phase on each cup. The cups were placed into NGI gentle rocker device and the cups were rocked for 5 min. The solution was collected from each cup and zolmitriptan content was determined through injection into the HPLC system. The in-vitro deposition profile of the prepared formulation was carried out in triplicate and the results were presented as the average results.

Fine particle dose (FPD), fine particle fraction (FPF), emitted dose (ED), and dispersibility were calculated as follows (Yazdani et al., 2014; Simona et al., 2016).

Fine particles dose (FPD) = mass of particles on stages 2 through 7

Fine particle friction (FPF) = fine particle dose/initial particle mass × 100.

FPF represents the percentage of emitted particles with an MMAD of 5 μm or less estimating the fraction of particles expected to deposit deep within the lungs.

Emitted dose (ED) = total particle mass on all stages/initial particle mass × 100

Dispersibility was defined as the ratio of FPF per emitted dose.

### 2.7. Physical stability

Physical stability of the selected MDI formulation (F1) was assessed by storing the formulation for 3 months at ambient temperature. After 3 months, the formulation was retested for its drug content per actuation, emitted shot weight, total number of discharges, leakage rat, moisture content, MMAD, GSD, FPF, ED, and dispersibility in the same methods just as mentioned above.

### 2.8. In-vivo pulmonary dosing of the selected zolmitriptan MDI formulation

For this study, animal handling was in accordance to the guidelines of the Research Ethical Committee of the National Organization for Drug Control and Research (NODCAR, Cairo, Egypt) and in accordance to the ethical procedures and policies approved by Ethical Research Committee of Faculty of Pharmacy, Cairo University, Cairo, Egypt and complied with the Guide for the Care and Use of Laboratory Animals (Institute of Laboratory Animal Resources (ILAR), 1996). For this study, adult male Sprague-Dawley rats weighing 200–210 g (*n* = 48) were divided into four groups. Animals were allowed free access to standard diet and tap water ad libitum and were housed at room temperature at natural light/dark conditions for 1 week before experiment. At the day of experiment, the animals administered to the tested treatments as follows:

Group 1: control which receives no medication.

Group 2: treated with the commercial tablets of zolmitriptan (Zomig^®^ 2.5 mg, astrazenica). The tablet was suspended into 1 ml saline and administered via gastric tube.

Group 3: received intravenous (iv) aqueous zolmitriptan solution (2.5 mg) injected through the tail vein of the rats.

Group 4: received the prepared zolmitriptan MDI, F1 (equivalent to 2.5 mg zolmitriptan). Administration was done using the method described by Sorkness et al. (2014). An infant MDI spacer chamber (Canack, Zhejiang, China (Mainland)) was used to allow delivery of an MDI aerosol to the awake rats by replacing the face mask and valve of the spacer chamber with a fenestrated latex membrane to serve as a nose cone for the rat. The rat was positioned in vertical position with only its nose and mouth within the chamber opposite the MDI canister. The MDI was actuated in the standard horizontal position, and then the chamber was rotated into a vertical position with the rat’s nose at the bottom. The rat was allowed to breathe from the chamber for 1 min after the actuation. Blood samples were collected at time intervals 5, 10, 15, 20, 30, 45, 60, 90, 120, 180, 360, 540, and 720 min into heparinized tubes, centrifuged at 3000 rpm for 15 min. The plasma samples were collected and kept frozen until analysis.

#### 2.8.1. Zolmitriptan HPLC chromatographic determination in plasma

Calibration curve of zolmitriptan in plasma, zolmitriptan extraction from plasma samples and its concentrations determination by a HPLC system were done using a previously described method (Awari et al., 2013; Abdou et al., 2017).

#### 2.8.2. Pharmacokinetic study

Concentrations of zolmitriptan in plasma samples were determined. Different pharmacokinetics parameters (*T*_max_, *C*_max_, and AUC) after iv, oral, and pulmonary administrations were calculated by WinNonlin software program (Ver. 1.5, scientific consulting Inc., Cary, NC, USA) by non-compartment analysis (Authier et al., 2016).

### 2.9. Statistical analysis

Data of all the experiments were expressed as the mean value ± SD. Statistical data were analyzed by one-way analysis of variance (ANOVA) and *p* < .05 was considered to be significant with 95% confidence intervals

## 3. Results and discussion

### 3.1. Particle size distribution of zolmitriptan micronized powder

**Figure 1.**
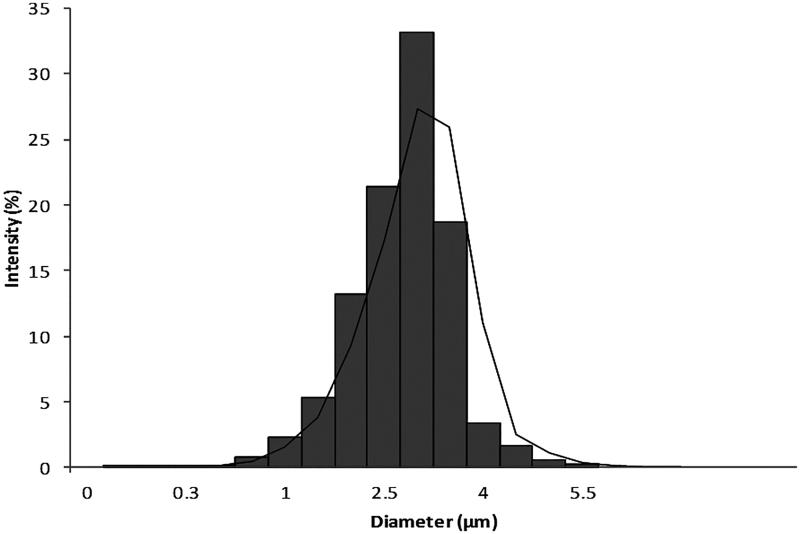
Micronized zolmitriptan powder particle size distribution.

Zolmitriptan particle size distribution measured through laser diffraction analysis is shown in Figure 1. The mean particle size diameter was found to be 3.25 µm while its median was 2.63 µm. *D* (10%) was 1.63 µm, *D* (50%) was 2.84 µm, and *D* (90%) was 3.95 µm to result in span of 0.47. These results suggest the possibility of using zolmitriptan micronized powder for MDI formulation as its diameter was less than 5 µm.

### 3.2. Development of HPLC method for zolmitriptan determination

For the developed HPLC method of zolmitriptan determination, the results of the system suitability parameters were 13064 for theoretical plates and 3.180 m as the retention time with 1.5 tailing factor and RSD of 1.4. The linearity was found in the range of 4–60 µg with correlation coefficient *R* ^2^ = 0.999. Results of recovery and precision studies are collected in Table 1. The method was found to be suitable, precise, and robust. The sensitivity of the method was determined based on the standard deviation of the response and the slope as described in ICH guidelines. The LOD and LOQ were found to be 1.56 and 4.74 µg/ml, respectively.

**Table 1.** Recovery and precision results of zolmitriptan determination HPLC developed method.

Accuracy (recovery studies)			Precision		
Concentration	Peak area	%	Concentration	Intraday (repeatability) (%RSD)	Interday (reproducibility) (%RSD)
15 µg/ml	25860.3	99.88%	35 µg/ml	99.88	99.63
25 µg/ml	47232.62	100.30%	45 µg/ml	100.31	100.50
35 µg/ml	68796.32	100.75%	55 µg/ml	100.75	100.89
	Mean	100.31	Mean	100.3133	100.34
	SD	0.432	SD	0.43501	0.645
	RSD	0.431	RSD	0.433651	0.643
	Variance	0.187	Variance	0.189	0.416

### 3.3. Evaluation of the prepared zolmitriptan MDI formulations

For MDI formulations, the suspended MDI formulations are usually restricted to be suitably shaken well just before use as to provide a controlled dose for the patient (Weers, 2000). Oleic acid was used as stabilizing agent to prevent irreversible particle agglomeration and drug particle adhesion to the container walls or the valve components. Also, it decreases the rate of separation between the drug and the propellant system and minimizes valve sticking problems (Myrdal et al., 2014).

Shot weights of different formulations, drug content per actuation, and the mean number of discharges per canister are listed in Table 2. In general, it is always required that the performance should be consistent and the MDI should deliver an accurate dose from the first until the last dose (Sukasamea et al., 2011). When the propellant HFA 134a having the higher vapor pressure (572 Kpa) (Zhu et al., 2014) was used (F1), the content of the drug delivered per one actuation was into the pharmacopeial limits (not less than 80% and not more than 120%). Also, there was nonsignificant difference (*p*-value>.05) between the actual content of the same formulation at different actuation times which confirms the uniformity of drug content till the last dose is actuated out of the canister. When the propellant HFA 227 having the lower vapor pressure (390 Kpa) (Zhu et al., 2014) was used (F2), the formulation drug content per actuation was lower than the accepted limits from the first to the last actuations. Using mixture of the two propellants (F3), having intermediate vapor pressure, the drug content per actuation was lower than the accepted limits at the first and the middle actuations and increased to be within the allowed limits at the last actuations indicating low drug content uniformity and non-ideal dispersion of the drug within the same canister. These results indicate that the difference in the vapor pressure of the propellant has significant effect on the drug content per actuation and drug content uniformity for the MDI formulations in accordance to what was previously described (Egla & Abd Al hammid, 2016).

**Table 2.** *In-vitro* evaluation of the prepared zolmitriptan MDI formulations.

	F1	F2	F3	Stored F1 (after 3 months)
Drug content per actuation (%)				
First	94 ± 8	58 ± 4	73 ± 6	99 ± 7
Middle	103 ± 11	64 ± 8	77 ± 10	95 ± 5
Last	97 ± 11	72 ± 8	85 ± 8	98 ± 9
Emitted shot weight (mg)				
First	58.9 ± 0.9	49.9 ± 2.4	49.3 ± 4.1	57.4 ± 1.3
Middle	60.4 ± 2.2	51.4 ± 2.4	53.8 ± 4.3	58.5 ± 2.1
Last	60.3 ± 3.2	50.4 ± 3.8	44.9 ± 4.2	58.1 ± 1.7
Total number of discharges	246 ± 17	248 ± 12	222 ± 15	241 ± 14
Leakage rat %	0.0022	0.0148	0.0114	0.0132
Moisture content (ppm)	172.0 ± 7.2	195.3 ± 5.9	182.7 ± 8.5	174.5 ± 5.3
FPF (%)	41.9	23.8	28.7	40.4
ED (%)	89.26 ± 2.35	55.21 ± 9.3	67.43 ± 2.84	90.62 ± 2.65
Dispersibility %	46.9	43.1	42.56	44.58
MMAD (µm)	3.47 ± 0.65	4.77 ± 0.45	4.1 ± 0.96	3.54 ± 0.72
GSD	2.3 ± 0.4	2.7 ± 0.6	2.9 ± 0.7	2.2 ± 0.7

### 3.4. Propellant leakage rate determination

The leakage rat of the tested formulations is listed in Table 2. According to the pharmacopeial limits which state that containers meet the requirements if the average leakage rat per year for 12 containers is not more than 3.5% of the net fill weight. None of the tested formulations was out of the limits.

### 3.5. Moisture content determination

Moisture content of the prepared formulations was found to be in the acceptable range, Table 2, which will not affect the product stability as it was reported previously that moisture content from 100 to 300 ppm (part per million) is very low level of moisture content while that above 300 ppm may influence the product stabilities (Cheng et al., 2001). Presence of water can greatly affect the product stability as water may initiate and enhance the hydrolytic degradation of the active constituent and may cause changes in particle size distribution or aggregation leading to reduction in the suspension homogeneity (Williams & Hu, 2000a). Furthermore, the moisture strongly affects the particle size distribution after actuation because it reduces the propellant evaporation rate and increases the apparent particle size (Murata et al., 2012) and consequently may affect the FPF of the formulation (Williams & Hu, 2000b). Therefore, water should be strictly limited to prevent these changes.

### 3.6. Microbial contamination determination

According to the pharmacopeial limits for inhaled products (Vu et al., 2014), all the prepared formulations were found to have an acceptable range of microbial content as the three formulations had both bacteria and fungi count less than 10 c.f.u./gm (c.f.u.: colony forming unit) with complete absence of both *P. aeruginosa* and *S. aureus*.

### 3.7. In-vitro aerosol deposition studies using the NGI

The NGI was originally introduced for the determination of the aerodynamic particle size distribution of the dose emitted from metered dose and dry powder inhalers using compendial procedures. It can categorize particles based on their size which is useful in predicting in-vivo deposition of particles. MMAD is one of the important factors that control deposition of particles in the different stages of the impactor and thus in the respiratory airways (Nahar et al., 2013). The amount of zolmitriptan from each formulation in each stage of NGI device was analyzed by HPLC system and the in-vitro deposition parameters including, MMAD, GSD, FPF, ED, and dispersibility were collected in Table 2.

The ideal size for a therapeutic particle is not known exactly, but it may be assumed that the MMAD should be not more than 5 μm to pass into the trachea-bronchial tree and smaller airways if peripheral deposition is required (Shi et al., 2009; Byron et al., 2010; Haddrell et al., 2017).

**Figure 2.**
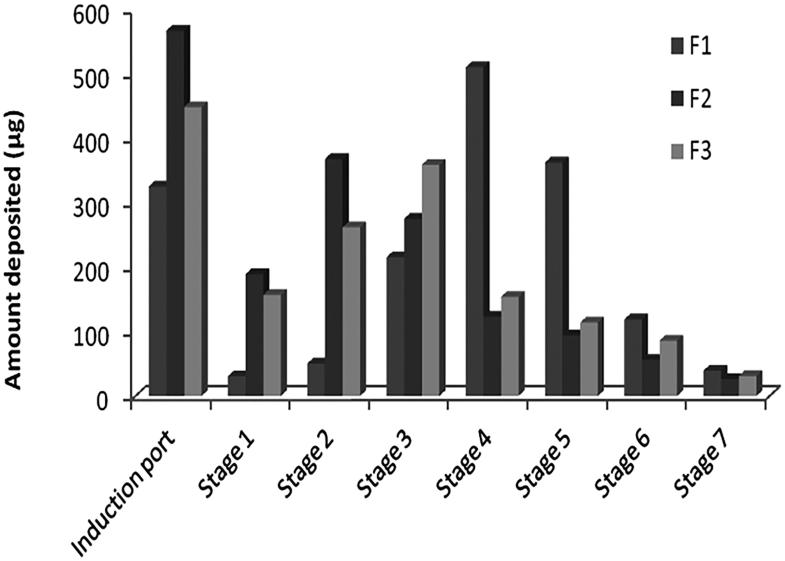
Zolmitriptan amount deposited into different stages of the NGI from different formulations.

Formulation F1 was found to have the highest FPF ratio, which represents the amount of drug that is considered respirable, indicating less deposition in the actuator, induction port, and stage 1 in comparison to higher deposition in other stages, Figure 2. On the other side, formulation F2 has the lowest FPF value indicating higher deposition in the actuator, induction port, and stage 1 rather than other stages, Figure 2. This can be explained by the higher vapor pressure of the propellant HFA 134a, used in F1, which provided a fine aerosol due to rapid propellant evaporation as the velocity of the plume discharge resulted in a large amount of drug deposited in the late stages while the lower vapor pressure of the propellant HFA 227, used in F2, resulted in insufficient vapor pressure for generating a respirable fraction (Egla & Abd Al hammid, 2016). This is the reason to find F3 has in-between FPF value due to use of mixture from the two propellants. Also, this relative high friction occurs in F1 resulted in significantly smaller MMAD than other formulations although the used active ingredient has the same particle size distribution. Depending on the above results, formulation F1 was selected for physical stability testing and in-vivo evaluation in rats.

### 3.8. Physical stability

An important concern for formulating suspension MDIs is instability due to non-ideal dispersion of the drug (O'Donnell & Williams, 2013). Comparing the stored F1 formulation with the fresh one resulted in no-significant difference was found in any of the evaluated tests, Table 2, indicating good physical stability of the suspension system and redispersibility of micronized zolmitriptan in the used propellant.

### 3.9. In-vivo pulmonary dosing of the developed zolmitriptan MDI formulation

**Figure 3.**
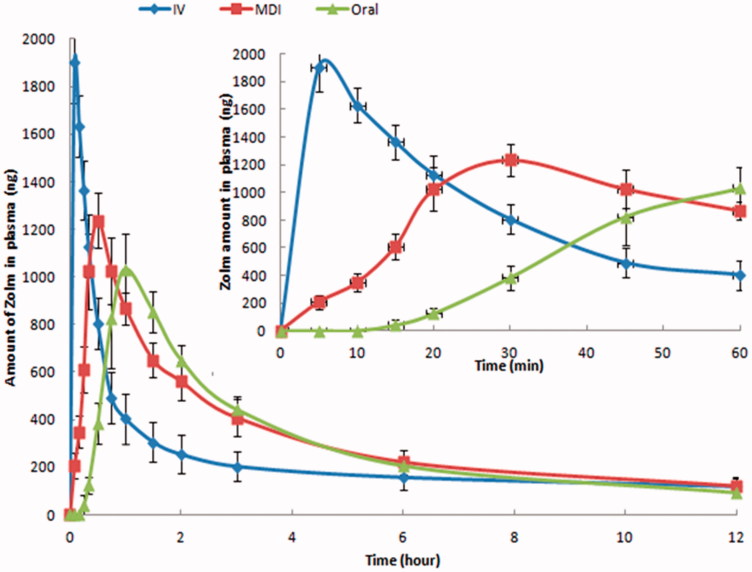
Zolmitriptan plasma concentrations in rats after administration of zolmitriptan MDI formulation, oral tablet, and i.v. solution.

Different pharmacokinetic parameters of MDI, iv solution, and commercial oral tablet of zolmitriptan in rats are collected in Table 3 and their time-plasma concentration profiles are shown in Figure 3. After zolmitriptan MDI formulation administration, zolmitriptan has appeared in plasma after 5 min for all the tested animals reaching its maximum at 27 ± 5 min while after zolmitriptan oral tablet administration, zolmitriptan appeared in plasma after 15 min for only two-thirds of the tested animals reaching its maximum at 53 ± 8 min. This indicates that MDI of zolmitriptan can provide faster onset of action over its oral tablets. Also, zolmitriptan MDI formulation has significant (*p*-value<.05) higher *C*_max_ and AUC_(0–12)_ over zolmitriptan oral tablets. This higher bioavailability of zolmitriptan MDI relative to its oral tablets indicates the ability of the formulation to provide the drug to the lower airways and bronchioles having thin linings, high surface area, and rich vascularization that allows rapid absorption of the medication and makes lungs an excellent portal for aerosolized medications intended for systemic absorption (Chua & Silberstein, 2016) in addition to low local metabolic activity in lungs and avoiding the hepatic first pass effect (Marianecci et al., 2011; Stank & Steckel, 2013). Relative bioavailability of zolmitriptan MDI formulation was 72.2% with 1.25 times higher than that of the oral tablet. This enhanced bioavailability was obtained through formulating the micronized zolmitriptan powder without further particle modification or engineering. Further studies using engineered zolmitriptan particles or drug-excipient formulation may result in more enhanced bioavailability that may sometimes approach 100% using the pulmonary rout (Patton & Byron, 2007). Enhancement of an anti-migraine drug (PCZ) through pulmonary inhalation in dogs and humans was previously reported by Rabinowitz et al. (2003). In addition, MAP0004, which is dihydroergotamine mesylate inhalation aerosol for acute treatment of migraine has clinically tested and found to be rapidly absorbed and have pharmacokinetic parameters similar to the iv solution of the drug (Silberstein, 2012).

**Table 3.** Pharmacokinetic parameters in rats after administration of zolmitriptan MDI formulation (F1), commercial tablet and iv solution.

	*C*_max_ (ng/ml)	*T*_max_ (min)	AUC _(0–12)_ (ng·min/ml)	Relative bioavailability
Zolmitriptan iv solution	1904 ± 174	–	4638 ± 634	–
Zolmitriptan MDI	1236 ± 116*^#^	27 ± 5^#^	3375 ± 482*^#^	72.7^#^
Zolmitriptan oral tablet	1033 ± 146*	53 ± 8	2698 ± 381*	58.2

* Significant from the i.v. solution (*p*-value<.05).

# Significant from the oral tablet (*p*-value<.05).

## 4. Conclusion

Zolmitriptan was formulated as MDI using different propellants. All the formulations showed accepted limits of leakage rate, moisture content, and microbial content. The formulation (F1) formulated using HFA 134a propellant showed accepted limits of shot weight and drug content. When it was tested for its in-vitro deposition using NGI apparatus, it showed higher deposition over other formulations into the stages 2–7 which represent the lower airways and bronchioles. The main factor affected the physicochemical properties of the tested formulations was the vapor pressure of the used propellant. The selected formulation (F1) showed higher bioavailability in rats over zolmitriptan commercial oral tablet. Unlike the common use of MDI formulations in treatment of asthma, micronized zolmitriptan MDI formulation using HFA 134a propellant could be a suitable dosage form for migraine treatment with enhanced bioavailability and faster onset of action. Further studies using engineered drug or drug-excipient particles in presence of different propellants ratios should be done in order to investigate other formulations with more enhanced in-vitro and in-vivo properties.
